# Physiological Perspective on Therapies of Lymphatic Vessels

**DOI:** 10.1089/wound.2017.0768

**Published:** 2018-07-01

**Authors:** Witold W. Kilarski

**Affiliations:** Institute for Molecular Engineering, The University of Chicago, Chicago, Illinois.

**Keywords:** lymphangiogenesis, hyperplasia, lymphedema, VEGF-C, wound healing, islets transplantation

## Abstract

**Significance:** Growth of distinctive blood vessels of granulation tissue is a central step in the post-developmental tissue remodeling. Even though lymphangiogenesis is a part of the regeneration process, the significance of the controlled restoration of lymphatic vessels has only recently been recognized.

**Recent Advances:** Identification of lymphatic markers and growth factors paved the way for the exploration of the roles of lymphatic vessels in health and disease. Emerging pro-lymphangiogenic therapies use vascular endothelial growth factor (VEGF)-C to combat fluid retention disorders such as lymphedema and to enhance the local healing process.

**Critical Issues:** The relevance of recently identified lymphatic functions awaits verification by their association with pathologic conditions. Further, despite a century of research, the complete etiology of secondary lymphedema, a fluid retention disorder directly linked to the lymphatic function, is not understood. Finally, the specificity of pro-lymphangiogenic therapy depends on VEGF-C transfection efficiency, dose exposure, and the age of the subject, factors that are difficult to standardize in a heterogeneous human population.

**Future Directions:** Further research should reveal the role of lymphatic circulation in internal organs and connect its impairment with human diseases. Pro-lymphangiogenic therapies that aim at the acceleration of tissue healing should focus on the controlled administration of VEGF-C to increase their capillary specificity, whereas regeneration of collecting vessels might benefit from balanced maturation and differentiation of pre-existing lymphatics. Unique features of pre-nodal lymphatics, fault tolerance and functional hyperplasia of capillaries, may find applications outreaching traditional pro-lymphangiogenic therapies, such as immunomodulation or enhancement of subcutaneous grafting.

**Figure:**
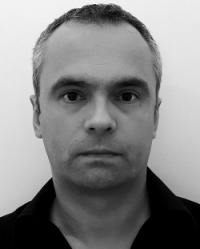
**Witold W. Kilarski, PhD**

## Scope and Significance

Recent discoveries about the physiological functions of lymphatic vessels and the identification of markers and growth factors associated with lymphatics have changed the perception of this auxiliary circulation. However, multiple knowledge gaps persist, which undermine the informed design of treatments, even for disorders such as lymphedema, which arises from impaired lymphatic drainage. In this review, I focus on the role of the endothelium in capillary and collecting lymphatic vessels, two structurally and functionally distinct compartments of the lymphatic system. From this perspective, I summarize the preclinical results from vascular endothelial growth factor (VEGF)-C pro-lymphangiogenic therapies, with an emphasis on prospective therapeutic concepts.

## Translational Relevance

Restricting the dose or the exposure time of VEGF-C reduces its pluripotent effect on blood vessels,^1,2^ the complication that limited applications of pro-lymphangiogenic therapies.^3,4^ A controlled administration of VEGF-C^5^ can also alleviate the risk of abnormal valve development within collecting lymphatics,^6^ preventing malformations that can cripple the drainage from the entire afferent lymphatic vasculature.^7^ In addition, research is revealing a growing list of variables, such as the subject's age^5^ and the presence of inflammatory stimuli,^8,9^ that can influence the VEGF-C treatment. Understanding the interplay between factors involved in non-developmental remodeling of lymphatics requires the generation of animal models that permit functional distinction between lymphatic capillaries and collecting lymphatic vessels in different tissues.^10^

## Clinical Relevance

Use of growth factor therapies is limited by undesired side effects arising from excessive or off-target responses to factors administered at high concentrations.^11^ Because of the relative infancy of the discipline, basic pro-lymphangiogenic research has not been clinically verified, and safety results from the phase I clinical trial of the first-in-man pro-lymphangiogenic Lymphactin^®^ (Herantis Pharma) therapy are not anticipated until 2019.^12^ However, analysis of the ambiguous and complex effects of VEGF-C in animal models can reveal obstacles of future clinical trials.

## Contrasting Lymphatic Functions and Pathologies

The open circulation of the lymphatic system is composed of capillaries, collecting vessels, and functionally distant, lymph node sinuses.^13^ The primary role of lymphatic capillaries and collectors is to transport tissue fluid, macromolecules, and immune cells from the interstitium through the lymph nodes and back to the blood circulation. Ontologically, lymphatics are derived from the venular compartment of blood circulation, which is reflected by the shared expression pattern of proteins that are essential for common functions of blood and lymphatic vessels: transport of fluid, the attraction of leukocytes, and assistance in their adhesion and transmigration. At the same time, the physiology and the biological relevance of blood and lymph circulatory systems differ drastically. For example, blood vessels are exposed to possibly the highest concentration of oxygen and nutrients, whereas hypoxic lymphatics are submerged in the effluent of tissue metabolites. Undoubtedly, lymphatics play a minor role in animal survival and even damage to the thoracic duct, the largest of lymphatic vessels, does not pose an imminent life threat.^14^ As a result, injured lymphatics are not compelled to patch hemorrhages as vigorously and consistently as blood vessels do,^15^ which justifies the absence of thrombocytes, a cellular arm of the blood clotting mechanism, in the lymph. Further, in contrast to blood vessels where control of blood fluidity is recognized as a factor that determines their functionality, we have only scant knowledge about mechanisms employed by the lymphatic endothelium to control lymph hemostasis in normal or pathologic conditions.^16^ This example illustrates how incomplete is our understanding of the fundamental biology and, in consequence, pathology of the lymphatic system.

### Lipid transport

Clinical research has extensively explored the transport of dietary lipids, lipid-soluble vitamins, and hydrophobic drugs from the small intestine.^17–19^ As a consequence, the lymphatic network of the intestine is the most studied lymphatic compartment.^20,21^ Within the intestinal villi, triglycerides are encapsulated by enterocytes into large (100–600 nm) chylomicrons and absorbed into the lacteals.^22^ Chylomicrons are transported via collecting mesenteric lymphatics to the thoracic duct and released into the left subclavian vein, thus circumventing the first-pass liver metabolism.^18^ In cases of congenital abnormalities or injury to the central lymphatic vessels, leakage of lymph from the lymphatic duct causes chylothorax^23^ or chylous ascites,^23^ the accumulation of lipid-rich lymph (chyle) in pleural or peritoneal cavities, respectively. Recently, lymphatic involvement in reverse cholesterol transport has also been described. Through this process, lymphatic capillaries participate in transcytosis and drainage of high-density lipoproteins (HDL, ∼10 nm) away from peripheral tissues, thus preventing interstitial accumulation and irreversible oxidation of cholesterol. Lymphatic control of HDL transport, in combination with the preferential binding of estrogens to HDLs,^24^ potentially explains the low levels of estradiol (but not progesterone) and pregnancy failure after the inhibition of follicular lymphangiogenesis with anti-VEGF-C antibody applied before the gestation.^25^ Lymphatic transport of estrogen bound to HDLs likely affects its pharmacodynamics as compared with estrogen transported in the blood when bound to albumin or its dedicated blood carrier, sex-hormone binding globulin. Verification of the effect of lymphatic density on systemic bioavailability of other steroid hormones, that is, corticosteroids, mineralocorticoids, and testosterone, could reveal new potential therapeutic targets in hormonal pathologies, such as Cushing syndrome or sterility caused by oligospermia. Nevertheless, a systemic link between the lymphatic transport of lipoproteins and human pathologies has yet to be established.^26^ That being said, the results of anti-lymphangiogenic therapies that target VEGFR-3 should be taken with caution as the expression of the VEGF-C receptor is not entirely specific to lymphatic endothelium, and its inhibition might influence vessels or structures not related to the lymphatic system.

### Immunity

The key immuno-related activities assigned to the lymphatic vasculature are concentrated in the endothelium of lymphatic capillaries. Capillaries permit the entry of interstitial fluid and dissolved macromolecules into the lymphatic lumen. Capillaries can also uptake and transport large extracellular vesicles, including exosomes (5–100 nm), microvesicles (100–1000 nm), and apoptotic bodies (>1000 nm).^27^ Endothelial cells lining these vessels secrete chemokines and assist in the entry and intralymphatic crawling^13,28^ of dendritic cells (DCs) and possibly T cells^29^ and neutrophils.^13,28,30^ Recently, lymphatic endothelial cells were shown to accumulate specific proteins from the interstitium^31^ and directly present antigens to T cells.^32,33^ Capillary lymphatics fuse into afferent (pre-) collecting lymphatic vessels, which then converge into larger, nonbranched collectors. Lymph within these terminal vessels is propulsed by the phasic contraction of autonomous contractile apparatus of lymphatic collectors, which has been described in detail in excellent reviews.^34,35^ Afferent collectors drain lymph into the subcapsular sinus of the regional lymph node, delivering collected tissue fluid with tissue antigens and passively flowing memory CD4^+^ and CD8^+^ T cells (80–90% of all cells from the lymph of the thoracic duct), DCs (5–15%), and B cells.^29,36^ In addition, all types of granulocytes and monocytes that can also present antigens to T cells drain to the lymph node during inflammation.^30^ Independently of the lymphatic transport of leukocytes, naive T cells, B cells, and monocyte-derived DCs enter lymph nodes through high endothelial venules of lymph node blood circulation.

The lymph node orchestrates a space for optimal interaction between antigen-presenting cells and lymphocytes, which result in mounting tolerogenic and adaptive immunity.^13^ If the lymphatic route is blocked, the immune system would be unaware of an inflammatory process occurring in the afferent tissue and remain unengaged, resulting in immune ignorance.^37^ Immune ignorance differs from immune suppression or tolerance because it preserves the existing state of immunity by blocking the first step of the immune response, the antigen recognition. Practically, blocking lymphatic drainage can be used to prevent rejection of small tissue allograft. This is possible due to a specific mechanism of systemic re-connection of tissue grafts, which, in contrast to organ transplants, are vascularized by blood vessels of the host. As a result, tissue implants lack the donor blood endothelium, a systemic source of alloantigens and a primary cellular target for alloreactive T cells and alloantibodies. In contrast, tissue grafts are placed within the interstitium with effective lymphatic drainage, which immediately delivers their alloantigens to the draining lymph nodes.^38^ Therefore, occlusion of lymphatic drainage from the allograft blocks the main route of alloantigen delivery to the immune system. Indeed, anti-lymphangiogenic therapy doubled survival of cornea grafts.^39^ However, it was a proximal lymphadenectomy (excision of lymph nodes) and lymphangiectomy (excision of collecting lymphatics), crude but the most definite approaches to lymphatic blockage, provided a proof of concept for the importance of lymphatics in allosensitization.^39–42^ Side effects associated with the invasiveness of this procedure exclude it from potential clinical application.^43^ In contrast, lymphatic-specific photodynamic therapy (PDT), a minimally invasive procedure with no adverse effects to the surrounding tissue, has the potential to immunologically seclude a fragment of the surface tissue, such as skin, and turn it into an immune-privileged site.^44^ Lymphatic-specific PDT uses the same compounds and activation techniques as standard PDT but differs from the classic approach in the administration route of photosensitizer (proto-toxin). In lymphatic-specific PDT, a photosensitizer (verteporfin) encapsulated in large 100 nm liposomes (Visudyne^®^) is injected locally into the dermis and is thereby collected and drained by lymphatic vessels. A laser beam (λ = 690 nm) is then applied downstream of the injection site and turns the verteporfin collected by the lymphatics into a self-destructing generator of toxic reactive oxygen species. Together with a short life-span of free radicals in oxygenized tissues that surround lymphatics, this setup assures specific and highly efficient decellularization of lymphatic collecting vessels. The decellularization triggers and sustains the lymphatic occlusion that blocks the tissue drainage of macromolecules to the local lymph node.^44^ The unexplained mechanism of occlusion by which decellularized basement membrane (BM) tubes of lymphatic collectors blocks the lymphatic drainage is likely the reason that lymphatic-specific PDT has not found widespread applicability.^44,45^ Nevertheless, decellularization of lymphatic collectors completely blocks migration of melanoma metastasis (hence possibly also immune cells) to the lymph node.^45^ Indirectly, the applicability of lymphatic blockage on graft survival has been tested with a standard PDT where intravenously injected photosensitizer blocked both blood and lymphatic vessels and delayed rejection of subsequent allograft^46^

It should be noted that the concept of transient occlusion of lymphatics as a physiological response to infection was proposed by Menkin almost a century ago.^47^ The author suggested that lymphatic occlusion could be a part of a defense mechanism intended to inhibit the systemic spread of the infection. Practically, ligation of lymphatic collectors in rabbit hindlimb stopped not only the systemic spread of bacteria but also protein toxins of snake venom, preventing death of experimental rabbits.^48^ Along these lines, lymphatic-specific PDT could be used to target localized threats, for example, to prevent infective larvae from reaching their final habitat during the early phase of asymptomatic filaria infection or as a means of blocking the systemic dissipation of microfilaria^49^ from lymphatic-dwelling adult worms.

### Fluid transport

Lymph nodes concentrate circulating lymph by extracting water through high endothelial venules.^50^ In the process, they integrate two key functions of the lymphatic system: immunity and fluid transport. In steady state, peripheral lymphatics drain the entire amount of fluid filtered by blood vessels.^51^ Chronic abnormalities in this fluid transport lead to the most well-recognized diseases of lymphatic circulation.^52,53^ In congenital and secondary lymphedema, the developmental abnormalities or injury to the collecting lymphatics lead to the accumulation of fluid and swelling of the limb, followed by irreversible fibrosis.^54^ Ocular hypertension in chronic (open-angle) glaucoma is caused primarily by occlusion of the trabecular meshwork^55,56^ as well as by Schlemm's canal,^57^ which are specialized vessels of the eye drainage system that have recently been classified as lymphatics.^58^ As much as trabecular channels can be considered capillaries, ocular hypertension is the only disease in which the initial compartment of lymphatics plays a significant role. This circumstance highlights the peculiarity of the lymphatic system, the diseases of which are almost entirely associated with collecting vessels.^59^ For example, the obstruction of drainage in lymphedema distichiasis is caused by valve abnormalities,^60^ with no role ascribed to lymphatic capillaries, whereas surgical excision of collecting lymphatics is a key but insufficient factor causing secondary lymphedema.^61^ The fault tolerance of lymphatic capillaries might be explained by their considerable plasticity and their ability to compensate for an increased fluid load, with a lymph flow rate that can be increased of an order of magnitude during inflammation.^62^

Some organs and systems, such as the central nervous system (CNS), bones and bone marrow, and placenta, are fully functional despite their lack of classic lymphatic drainage.^63^ Therefore, they must have alternative means of dealing with fluid leaking from blood vessels into the interstitium. The process of fluid accumulation in tissues is driven by an imbalance between the hydrostatic and colloid osmotic pressures of capillaries and the interstitium.^64^ Initially, net filtration can be inhibited by an increase in the hydrostatic pressure of interstitial fluid modified by fibroblast-mediated tissue contraction^64^ or by the release of intradermally stored osmotically inactive sodium.^65,66^ Nevertheless, the accumulated interstitial fluid must eventually be evacuated, as according to the revised Starling's model venous reabsorption does not play a significant role in returning plasma solute to the circulation under steady-state (homeostatic) conditions.^50,51^. The brain and the spleen exemplify opposing ways of dealing with this problem. In the CNS, blood capillaries are sealed with tight junctions that minimize the leakage of water and ions into the parenchyma.^67^ In contrast, in the spleen, a discontinuous blood endothelium and its BM allow blood to freely perfuse the tissue,^68^ which prevents the formation of an osmotic gradient. To some extent, lymphatics present in serous membranes surrounding the spleen^69^ or the dura matter^70^ can drain fluid and macromolecular antigens from organs deprived of lymphatics. Indeed, this ability was recently confirmed for the entire brain.^71,72^ However, the physiological relevance of lymphatic drainage of the cerebrospinal fluid is not clear because dura lymphatics do not participate in relieving the CNS of its interstitial fluid load.^73^ Instead, lymphatic drainage in the dura is speculated to be relevant for the development of an adaptive immune response in the CNS. However, although cerebrospinal fluid is in communication with the interstitial fluid of the brain parenchyma, these two liquids are not homogeneous, and cerebrospinal fluid cannot be assumed to be representative of the CNS interstitial fluid.^74^ Brain metabolites and antigens are transported via the interstitium to the cerebrospinal fluid^75^ during sleep through a glio-vascular pathway within paravascular spaces surrounding large veins.^76^ Therefore, lymphatics located within the dura collect antigens already diluted in cerebrospinal fluid. These antigens are also delivered to the lymph node long after their release from the brain parenchyma and exposure to proteolytic modification or complete digestion within the CNS.^77^ In addition, the lack of spatial and conditional resolution of the antigens' release sites complicates the development of opposing adaptive and tolerogenic responses.

The relevance of lymphatic circulation in organs with intrinsic lymphatic systems, such as pancreas, lungs, heart, liver, and kidney, is poorly studied,^52,78^ but the presence of such systems is generally regarded as expendable for the organs' core functions.^79^ However, with liver lymphatics (producing up to 50% of human lymph^80^) or the two independent (subcapsular and cortical) lymphatic systems of the kidney,^81^ we should acknowledge our ignorance rather than attribute an atavistic nature of these under-researched circulatory systems. Indeed, major organs can function without active lymphatic drainage. For example, before heart or kidney transplantation, lymphatic vessel connections between the graft and the recipient are left disconnected or are purposely ligated.^82,83^ In a minority of cases, this treatment might lead to lymphorrhea and lymphocele around the transplanted organ, but, in general, it has no significant detrimental effect on its function.^84,85^ In contrast, lymphatic occlusion in limbs might eventually produce lymphedema, chronic edema consolidated by subsequent tissue fibrosis. Even then, however, the etiological role of lymphatics remains unclear. For example, the reported incidence of edema after mastectomy varies substantially, from 6% to 80%,^86^ and its precipitating factors^61^ are not known.^87^

Secondary lymphedema also develops as a late complication of lymphatic filariasis, a parasitic disease with a complex and often misunderstood etiology.^49^ During the asymptomatic phase of the infection, larvae of parasitic filarial nematode escape the intermittent host, a mosquito, enter the skin, and migrate to the collecting lymphatics.^49^ During 10–15 years of their life within lymphatic collectors, matured filariae produce millions of microfilaria larvae, which are drained with the lymph to the blood circulation. The presence of adult filaria induces endothelial proliferation and lymphangiectasia,^88^ the likely changes in collectors that lead to lymph backflow,^89^ the characteristic sign of the disease. Nevertheless, the direct cause of eventual lymphedema is generally not the occlusion of collecting lymphatics by centimeter-long adult parasites. To the contrary, *Wuchereria bancrofti* or *Brugia* filarial worms actually stimulate lymph flow, and that sign is so characteristic for the filaria infection that it became part of the diagnosis in asymptomatic patients.^89^ Even ongoing deaths of adult worms causing acute filarial lymphangitis and the associated local injury to the vascular endothelium only transiently obstruct lymphatic flow.^16^ Instead, lymphedema and elephantiasis, a massive accumulation of fibrous tissue in the affected limb, can develop after recurrent bacterial and fungal infections and associated dermatolymphangioadenitis. However, lymphedema can develop in individuals without evidence of any currently active infection or even lymphatic blockage, which further complicates pinpointing the complete etiology of this lymphatics disease.^49,90^

Lipedema is an idiopathic disease that is often discussed together with lymphedema likely due to their phenotypical similarities as there is no compelling evidence suggesting that lymphatic insufficiency could be a component of this pathology.^91^ Contrary, even in advanced lipedema, lymphatic drainage was mostly unaffected despite the presence of massive fibrotic changes in legs.^92^

The poorly understood etiology of lymphatic diseases correlates with unexpected difficulties in establishing a reliable lymphedema model that reflects the human condition with deposition of subcutaneous fibrotic tissue. Numerous physiological studies performed at the beginning of the 20th century were concluded in a Drinker and Field monograph.^93^ Authors expressed their frustration with futile attempts to establish an animal lymphedema model: “If it has been difficult to produce edema by venous stasis it has been impossible to produce edema, except for a short time by blocking lymphatics.” By that time, it was clear that a “second factor” is necessary for the induction of symptomatic disease.^90^ Indeed, an inflammation, a trigger of the disease that was earlier suggested by Manson-Bahr,^94^ played the sole causative role in the generation of lymphedema in the first animal model of the disease. Drinker, Field, and Homans showed that even without surgical ligation of collectors, multiple injections of silica and quinone directly in the lymphatic collectors were sufficient to induce lymphedema in a dog's hindleg.^95^ This treatment denuded lymphatic endothelial lining, whereas the ensuing foreign body reaction triggered chronic sterile inflammation that resulted in lymphatic fibrosis, development of persistent edema, and overgrowth of subcutaneous tissue.^78^ More recently, the surgical model of lymphedema in dogs was described by Olszewski.^96,97^ It closely mirrors the human disease, with symptoms appearing months or years after the surgical disruption of lymphatic collecting vessels. Similar to the model presented by Drinker at al., lymphatic collectors undergo fibrosis before the lymphedema of the limb develops. The unpredictable onset of lymphedema symptoms, however, complicates widespread deployment of this system, whereas in contrast to the inflammatory model presented by Drinker *et al.*, it also conceals the trigger of the disease.^59^ Inflammation is a component of the first available rodent model of lymphedema.^98^ Persistent swelling of the mouse tail develops after a combination of surgical excision of the full-thickness skin from the center of the tail and the inflammation that is inevitably fueled by the re-infection of the wound exposed to the cage environment and self-grooming. However, inflammation is a multifactorial process, and a plethora of various components might trigger and sustain lymphedema. Recently, leukotriene B4 was identified as a two-faced factor in lymphedema pathology. Pro-lymphangiogenic at physiological levels, it inhibits lymphangiogenesis and lymphatic repair at a high concentration.^8^ Other inflammatory mediators, such as TNFα^9^ and interleukin-1β,^99^ were found to promote steroid-resistant^100^ lymphangiogenesis and lymphatic remodeling. A superimposition of excessive remodeling of lymphatic and vascular hyperpermeability, both induced by Il-1β and TNF-α,^101^ might result in persistent fluid accumulation. Finally, rapamycin, an mTOR inhibitor and clinically approved IL-2-dependent immunosuppressant, reversed VEGF-C-induced lymphangiectasia,^102^ potentially linking lymphedema-related abnormalities with systemic immunity.

The disease remains a mystery because of an incomplete understanding of the biology and pathophysiology of lymphatics. Paradoxically however, because of a relative functional resistance to injuries or morphological abnormalities, the lymphatic system is less of a risky target for lymphatic-specific manipulation (discussed in the Contrasting Lymphatic Functions and Pathologies section).

## Contrasting Functions of Capillary and Collecting Lymphatic Vessels

**Figure 1.**
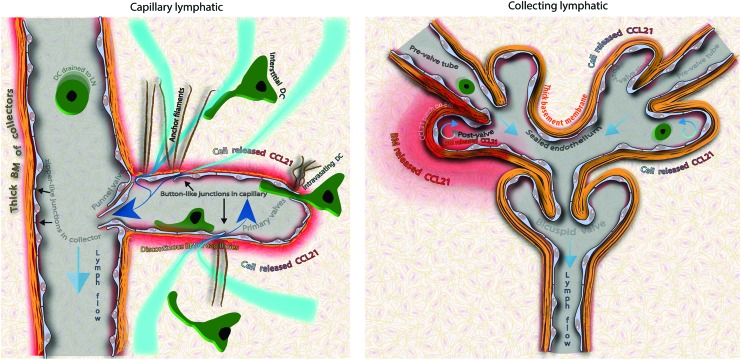
The different roles of capillary versus collecting lymphatics. *Left panel:* A schematic of lymphatic capillary connected to the pre-collecting lymphatic with a funnel-like valve.^109^ Capillary lymphatic vessels permit the flow of interstitial fluid and solutes into their lumen. Discontinuous junctions between lymphatic capillary endothelium (primary valves), together with pre-formed portals formed within their thin basement membrane, facilitate entry of interstitial fluid and leukocytes into the capillary lumen. The basal surface of endothelial cells is anchored to the surrounding matrix by approximately 10-μm-long and 4–15-nm-thick fibrillar filaments.^167^ These structures facilitate the opening of the primary valves in response to swelling-driven expansion of the tissue. Capillary endothelium produces chemokines that attract leukocytes expressing the CCR7 receptor. CCL21 chemokine stored in the endothelium is inaccessible until its release from the cell. Even then, it is not immediately available to memory T cells or dendritic cells as it binds to heparan sulfate proteoglycans, proteins that build the basement membrane. In conjunction with physical properties of the interface between interstitium and the capillary lumen, such as the void space within lymphatics, heterogenous basement membrane, and varying intensity of interstitial currents, the complex bio-accessibility of CCL21 allows generation of dynamic gradients, both outside and within the lymphatic capillary. Leukocytes enter the capillary lumen through the discontinuity of their thin basement membrane and flaps of the button-like junctions and crawl along the apical side of the endothelium until they reach the collecting vessel. In addition, lymphatic endothelial cells can actively transport protein, participate in the reverse cholesterol transport, and present antigens to T cells (not shown). *Right panel*: A schematic of lymphatic collecting vessels. The maintenance of the unobstructed and unilateral lymph transport within non-permeable lymphatic tubes is the primary function of collecting vessels, reflected in the unique morphology of their bicuspid valves.^111^ A narrow lymphatic tube ends within a larger post-valve sinus with two cuspids that are capable of sealing the lumen. The post-valve sinus helps to sustain the backflow by redirecting the force of the fluid during retrograde lymph movement, closing the valve as soon as the pressure in the efferent vessel dominates. Bicuspid valves are formed along the lymphatic collector (lymphangions) and at every junctional connection between converging lymphatic branches. In contrast to lymphatic capillaries, floating immune cells have limited contact with the endothelial lining whereas zipper-type junctions between the endothelial cells of collecting vessels restrict the fluid permeability. At random locations, the basement membrane of the collector accumulates large quantities of heparan sulfate-binding chemokine CCL21. Because leukocytes cannot intravasate through the wall of the collecting vessel, the role of these extracellular CCL21 stores is unclear. BM, basement membrane; DC, dendritic cell. To see this illustration in color, the reader is referred to the web version of this article at www.liebertpub.com/wound

The characteristic morphology of cell-cell junctions in the initial lymphatic capillaries^103,104^ gives them the ability to passively permit entry of large molecules such as extracellular vesicles or erythrocytes.^10,105^ Endothelial cells of lymphatic capillaries are docked to extracellular collagens by anchoring filaments.^106^ As the interstitial pressure rises and the tissue expands, these cell anchors stabilize capillaries, which would otherwise collapse with only the support of a thin and discontinuous layer of BM.^107,108^ In contrast, increased interstitial fluid pressure expands the surrounding matrix, and by pulling the anchors, opens “button-like”^103^ primary valves (∼3 μm in length and ∼0.5 μm in width) by creating discontinuities of VE-cadherin adherens junctions and thereby allowing interstitial fluid to enter the lumen of capillary lymphatics.^103^ Therefore, the functions of capillary lymphatics—permeability to interstitial fluid and ability to attract leukocytes—depend on the capability of individual endothelial cells to form correct intracellular and matrix junctions and release chemokines (Fig. 1, left panel).

In addition to the junctional arrangement at the cellular level, the function of lymphatic collectors depends on the higher-level organization through cooperation among numerous endothelial cells (Fig. 1, Right). The endothelium of collectors forms zipper-like junctions that seal the space between cells and limit vessel permeability.^103^ Intraluminal funnel-like valves with circumferential cuspid determine the direction of flow between capillary and pre-collecting lymphatic vessels.^109,110^ Bicuspid valves are present at every lymphatic bifurcation in larger collecting vessels, and along the pre-nodal nonbranching lymphatic collectors,^111^ where they outline lymphangions, the contractile units of collecting vessels.^35,112^ BM mechanically reinforces valve cuspids but also delineates the bulging morphology of post-valve sinuses.^113^ Sinuses help sustain the backflow during retrograde lymph movement by shifting a hydrostatic force laterally toward valve cuspids, facilitating closure of valves as soon as the pressure in efferent vessels dominates. Cuspids and sinuses of a funnel or bicuspid valves amount to the most characteristic morphological features of the lymphatic collecting vessels^108,114,115^ that are recognizable under a stereomicroscope in live, unstained tissue and even as decellularized casts of BM.^108,109^

**Figure 2.**
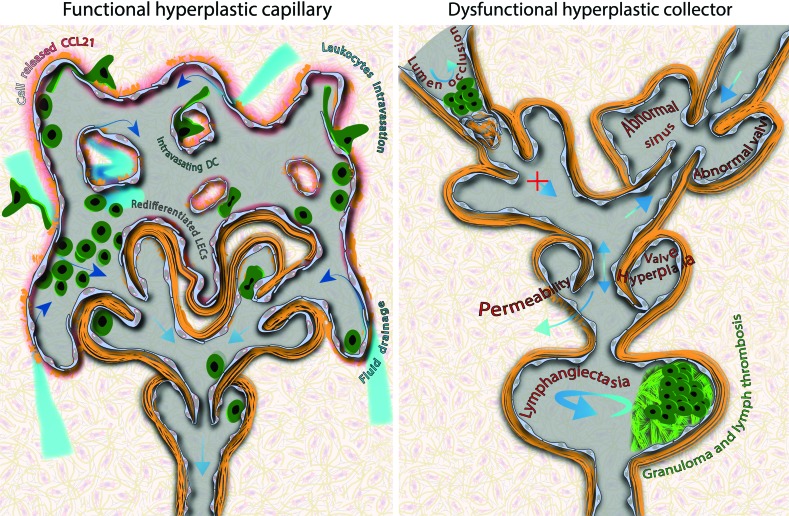
Hypothetical results of hyperplasia on the function of capillaries and collecting lymphatics. *Left panel*: The function of capillary lymphatics is determined at the cellular level. Despite abnormal morphology, hyperplastic capillaries remain functional as long as individual cells preserve their properties. A pre-requisite for the formation of a vessel, cell-cell junctions between endothelial cells, allows seclusion of the lumen by the intact monolayer of endothelial cells. Independently of vessel morphology, for the new capillary to remain functional, endothelial cells must secrete leukocyte chemoattractants, such as the CCL21 chemokine, to assist in leukocyte intravasation and preserve button-like morphology of primary valves. *Right panel*: The unidirectional lymph drainage along the collecting lymphatic depends on a structural organization of vessels and can be compromised even when individual cells preserve their properties. Hyperplasia affects the anatomical organization of multicellular structures and leads to the structural abnormalities in most hypothetical scenarios: drainage occlusion (intralymphatic proliferation), valve insufficiency and lymph backflow (valve remodeling), drainage block (formation of valves opposing the normal direction of lymph flow), lymph stasis, clotting, fibrosis (lymphangiectasia), and lymph leakage to the interstitium (increased permeability of endothelium). Also, in contrast to lymphatic capillaries, a single morphological aberration within lymphatic collectors may result in dysfunctionality of the entire afferent lymphatic drainage system. To see this illustration in color, the reader is referred to the web version of this article at www.liebertpub.com/wound

**Figure 3.**
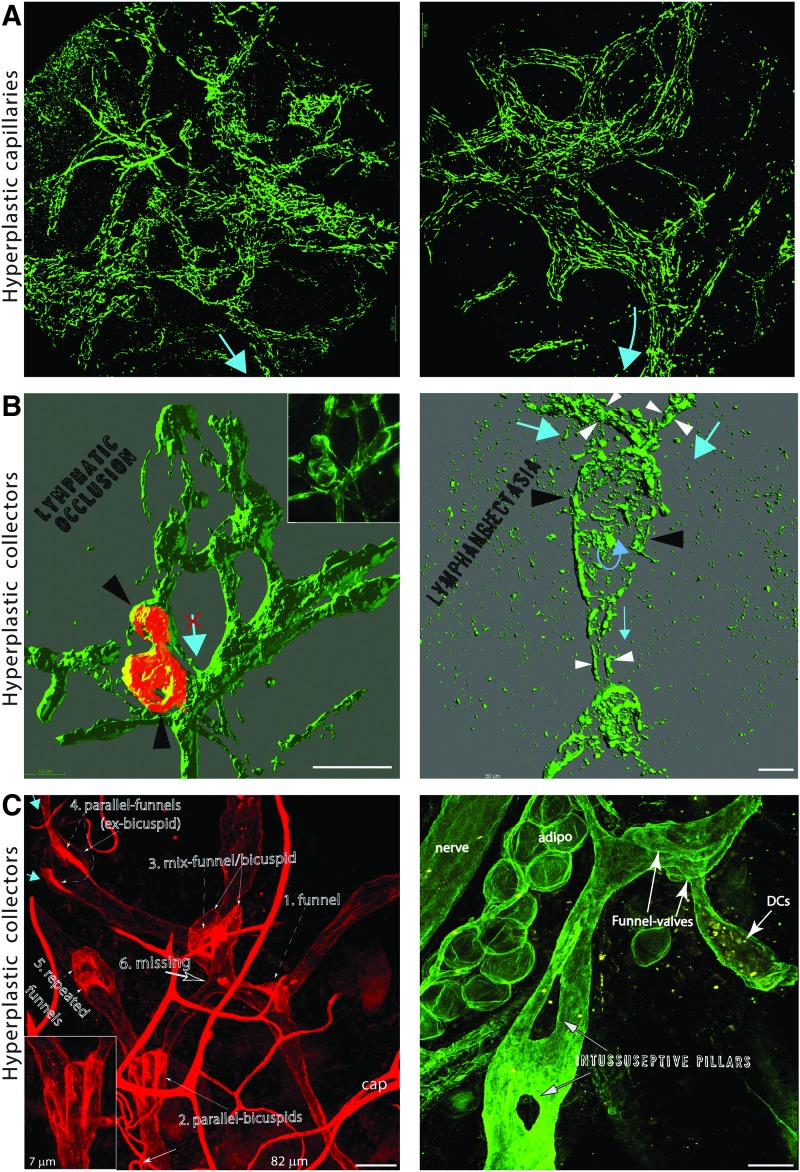
Results of hyperplasia on the function of capillaries and collecting lymphatics. **(A)** Hyperplastic capillary lymphatics (LYVE1, *green*) in the VEGF-C-treated wound. Despite the morphological abnormalities, these structures are potentially capable of draining fluid and leukocytes as they are connected to the central lymphatic circulation (*blue arrows*). (**B, C)** Hyperplastic abnormalities in lymphatic collecting vessels in the VEGF-C-treated wound. Dermal collectors were identified by their characteristic morphological feature, the: uneven diameter along the vessel length. **(B)** The variable signal of LYVE1 expressed by VEGF-C-stimulated collectors was uniformly masked during image post-processing **(B),** *left panel*. Occlusion of the lymphatic vessel (*black arrowheads*) by intralymphatic hyperplasia (LYVE1, *red*), which occupies the entire lumen of the collecting vessel. The *inset* shows the unmasked maximum intensity projection of the LYVE1 signal. **(B),** *right panel*. Lymphangiectasia of the lymphatic collector, a local increase in vessel diameter (*black arrowheads*) as compared with the afferent and efferent vessel (*white arrowheads*). The resulting lymph stasis might lead to lymph clotting, fibrosis, and, in consequence, a lumen occlusion. **(C),** *Left*. Abnormal growth of hyperplastic valves. CD31 staining of the network of the pre-collecting lymphatics that underwent hyperplastic remodeling in the VEGF-C-treated wound. 1. Funnel. A conventional, funnel-like valve (dashed *arrow*), generally found at the outlets of capillary and initial pre-collecting vessels, is correctly located at the junction of two pre-collecting vessels. Other valves show various morphological abnormalities. 2–4. Dependent (joined) valves. 2. Parallel-bicuspid. Two bicuspid valves (*arrows*) are abnormally fused: the cuspid of the left valve acts as the sinus wall of the right valve (see *inset*). 3. Mix-funnel/bicuspid. A funnel-like valve formed within another bicuspid valve. 4. Parallel-funnel. Two symmetrically oriented funnel-like valves draining two separated vessels (*blue arrows*) formed within a single bicuspid valve. 5. Repeated funnels. A redundant funnel-like valve formed at the outlet of the afferent one. 6. Missing. A missing valve between the *left* and the *right* fragment of the lymphatic network. Values in μm denote the thickness of the optical section reconstructed from consecutive confocal scans; cap-blood capillaries. **(C),** *right panel*. Staining for the basement membrane components reveals persistent structural changes to the circulation. A thick basement membrane demarcates lymphatic capillary enclosing a group of dendritic cells. Hyperplasia in collecting lymphatics resulted in a double intussusceptive split of the collecting vessel. Formation of intravascular pillars that split vessels is a sign of intussusceptive vessel growth.^168^ Note the absence of valves that should control the flow of the lymph at the bifurcation points (the beginning and the end of each pillar). *Blue arrows* point the direction of flow inferred from the morphology of the valves. Bars = 50 μm. VEGF, vascular endothelial growth factor. To see this illustration in color, the reader is referred to the web version of this article at www.liebertpub.com/wound

Discussed in detail in the following section, a therapeutic induction of post-developmental lymphatic remodeling comes with the risk of excessive stimulation of targeted cells that leads to their hyperplastic outgrowth. Even though unlike neoplasms, hyperplastic cells preserve their lineage-specific properties, changes to the tissue architecture could jeopardize its morphology-dependent properties.^116^ The hyperplastic growth of capillary endothelium poses a minimal threat to the capillary function as, despite spectacular morphological abnormalities,^10,117^ their function is determined at the cellular and intracellular levels (Figs. 2 Left panel and 3A). Therefore, hyperplastic capillaries should remain functional as long as individual cells preserve their physiological properties. To the contrary, excessive proliferation of endothelium within valves or lumen of collecting lymphatics could be detrimental for the unidirectionality of lymph flow and entirely block the drainage from the afferent tissue (Figs. 2 Right and 3B, C). Indeed, abnormal valve development is responsible for the insufficient lymph transport in hereditary lymphedema distichiasis^60^ and for the reversed lymph flow from solid tumors.^6^ Therefore, in sharp opposition to capillary lymphatics, morphological features of lymphatic collectors are indispensable for their functionality. Any abnormalities in cell arrangement or organization of their BM reflect the functional state of collectors that could serve as an indicator of their physiological status (Figs. 2 and 3).

**Figure 4.**
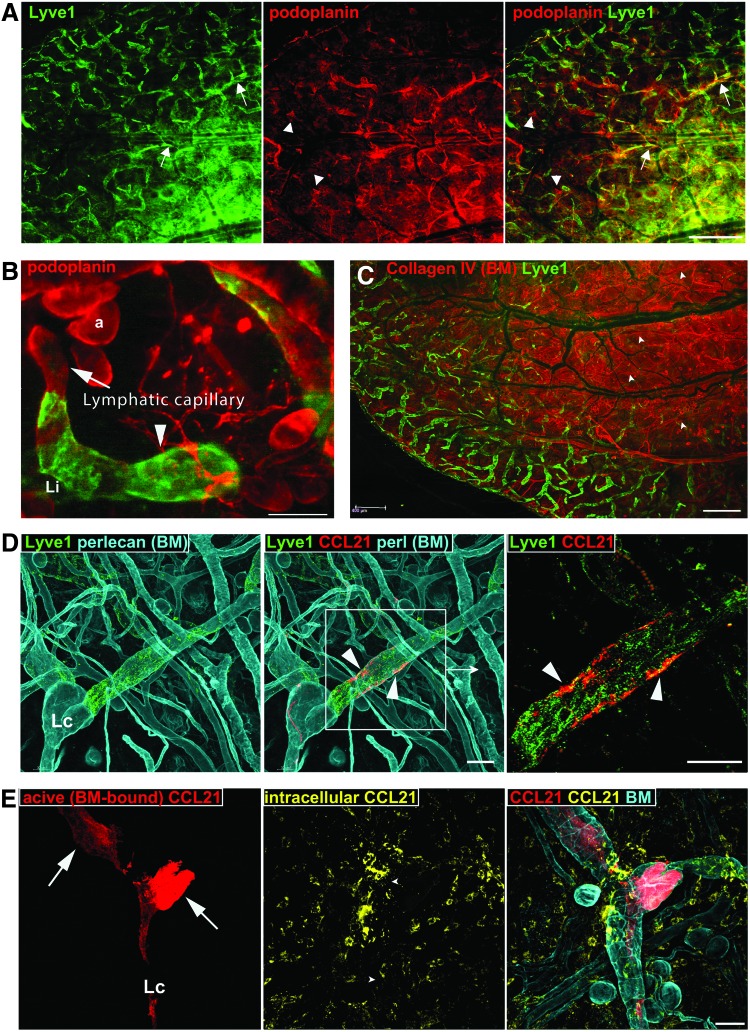
Heterogeneous expression of lymphatic markers and chemokines. **(A)** Fluorescent image of the lymphatic circulation in mouse dorsal skin of the untreated ear (approximately 40% of the dorsal ear surface is shown). LYVE-1 is expressed mostly by lymphatic capillaries in the outer fragment of the skin flap (*left panel*). Podoplanin is expressed mostly by collectors (*center panel*). Both stainings overlap, with podoplanin exclusively staining some of the lymphatic capillaries (*arrowheads*) and LYVE-1 staining the collectors (*arrows*). In addition, neither staining is continuous, with patches of cells negative for podoplanin or LYVE-1 in main clusters of predominantly LYVE-1 and podoplanin-positive cells. **(B)** Heterogeneous expression of LYVE-1 in two connected skin lymphatic blunt-ended capillaries. In contrast to the lower capillary (*arrowhead*), the top capillary enclosed within the basement membrane (BM) capsule (*arrow*) has patchy, all-or-none expression of LYVE-1.^108^ **(C)** Epifluorescent image of the dorsal skin of the normal ear live-stained for collagen IV (BM) and LYVE-1. LYVE1-negative collecting lymphatics are connected to the LYVE1-positive vessels. Collecting vessels can also be independently identified by their unique features reflected in their thick basement membrane: an uneven diameter along the vessel length, with the bulky sinuses of vessels at the valve outlet (efferent side of the valve, *arrowheads*). **(D)** Epifluorescent image of live dorsal skin from the untreated ear live-stained for CCL21 (*red*), perlecan (heparan sulfate proteoglycan of BM, cyan), and LYVE1 (*green*). Only extracellular (accessible) BM deposits of CCL21 are stained (*arrowheads*). **(E)** (*Left panel*) Epifluorescent image of the dorsal skin of the untreated ear live-stained for CCL21 (*red*), and perlecan (BM, cyan) shows the extracellular deposits of CCL21 on BM (*arrows*). (*Middle panel*) Post-fixation staining for CCL21 of the same skin additionally revealed inaccessible intracellular stores of CCL21 (spotted perinuclear rings, *yellow*). In addition to lymphatic endothelium, nonendothelial stromal cells produce and store CCL21. For clarity, the dominant signal from extracellular CCL21 was removed during image processing. (*Right panel*) Co-localization of BM (perlecan) staining with extracellular BM-deposited CCL21 (*red*) and inaccessible intracellular stores of CCL21 (*yellow*). Lc = location of lymphatic collector, a = adipocyte. Bars: A, C = 1000 μm, B, D, E = 50 μm. To see this illustration in color, the reader is referred to the web version of this article at www.liebertpub.com/wound

Advances in molecular profiling have revolutionized research in the lymphatic field by allowing histological identification of atypical lymphatic vessels and capillaries, for example, around solid tumors.^118^ However, lymphatic-associated receptors and transcription factors are also expressed in various other tissues. For example, a Prox1 transcription factor that determines the lymphatic profile of the endothelium has unknown function^119^ and is also expressed in pancreas, liver, and hypothalamus.^120^ In lymphatic collecting vessels, FOXC2 regulates all steps of valve formation, but it is also essential in adipocyte metabolism and the differentiation of kidney podocytes.^121^ Podoplanin is a membrane receptor expressed by lymphatics (Fig. 4A–C), and its interaction with platelets initiates separation of lymphatic and vascular circulation in the developing embryo.^122^ However, the function of podoplanin in the adult lymphatic endothelium, as well as in the epithelium, kidney podocytes, and astrocytes is not understood.^123^ LYVE-1, a hyaluronan receptor, is expressed by most capillary lymphatics (Fig. 4A–C), but it is also abundant on regeneratory M2 macrophages.^124^ After its discovery, the role of LYVE-1 remained elusive for 18 years, and its expression was only recently linked to DC trafficking, specifically the attachment of DCs to the lymphatic wall before their intravasation into the vessel lumen.^125^ Surprisingly, however, knockout of LYVE-1 has no effect on DC transmigration or mouse development.^126^

In contrast, lymphatic secretion of chemokines, that is, CX3CL1, CXCL12, and particularly CCL21, is essential for guiding leukocytes during their migration from the dermis to the lymph node.^127^ Concentrated stores of chemokines in the perinuclear Golgi apparatus of various cells are readily detected in histological or whole-mount preparations.^128^ However, because these chemokines are stored intracellularly, they remain inaccessible (biologically inactive until released by the cell) and can merely confirm the functional potential of the endothelium. Among the lymphatic-derived chemokines, CXCL12 and CCL21 have heparan sulfate proteoglycan (HSPG)-binding properties,^127^ and therefore have the capacity to form extracellular, and thus biologically active (availability is passively controlled by their dissociation constants), chemokine deposits (Fig. 4D, E). Specifically, they can bind to an extracellular HSPG domain of ubiquitous syndecan receptors or create extracellular stores on the HSPGs (perlecan, agrin, and collagen XVIII) of the BM^129^ On the one hand, the BM acts as a filter and prevents chemokines diffusion into the interstitium, which sequesters their activity within the lymphatic space. Indeed, intralymphatic gradients of CCL21 were found to guide DC intraluminal migration from capillaries to collecting vessels.^28^ On the other hand, the BM of lymphatics that accumulate CCL21 and CXCL12 can form a persistent and steep chemokine gradient outside lymphatic vessels. We found patches of stored CCL21 within the BM of collecting lymphatics that were able to attract interstitial DCs and neutrophils^108,130^ (Fig. 4D–F). In addition, CCL21 deposits are located within the patches of collecting lymphatics expressing LYVE-1 (Fig. 4D), which suggests the convergence between the BM-emerging CCL21 gradients and hyaluronan-LYVE-1–mediated docking of DCs to the lymphatic endothelium.^125^ This hypothesis, however, requires further experimental verification. The function of extracellular chemokine stores is unknown, but their relevance was questioned in a report in which authors used heparinase to dismantle BM and CCL21 binding sites.^131^ However, this study failed to identify the sparse, patchy staining of CCL21 on untreated BMs. The staining pattern of chemokines in unfixed tissue is morphologically distinct from the punctuate perinuclear staining of CCL21 as shown by our group (Fig. 4E).^108,130^

Lymphangiography, a minimally invasive functional test of lymphatic collectors, relied on injection of the tracer in the interstitium and is applicable only to live individuals or intact organs.^10^ In fixed tissues, however, the functional status of collectors can be inferred from their morphology, as outgrowth within the endothelial lining or excessive BM deposits results in lumen occlusion or valve hypertrophia, the abnormalities that can block the drainage from the entire afferent vasculature (Figs. 2 Right and 3B, C). It should be noted that tissue morphology cannot be directly retrieved from mechanically processed samples, that is, after the most common, mechanical sectioning of the tissue. Also, the presence or absence of lymphatic-associated markers such as LYVE-1 or podoplanin is heterogeneous (Fig. 4A, B) and indifferent to the function of collecting lymphatics.

Performance of capillary vessels is resistant to morphological abnormalities and cannot be inferred from capillary morphology (Figs. 2 Left and 3A). Acting at the cellular level, LYVE1 and junctional proteins, secreted chemokines, and BM components, together with the extracellular fibrilin anchoring filaments,^130^ determine the permeability and immune-related properties of lymphatic capillaries. In contrast to collectors, functional parameters of capillary lymphatics could not be directly assessed *in vivo* with a method such as a lymphangiography as the non-physiological pressure of injection artificially forces particles or cells into lymphatics. However, combining implantation models with an intravital imaging technique, we showed that morphologically abnormal hyperplastic capillaries perform two of their primary functions, draining macromolecules and attracting DCs and assisting in their intravasation.^10^

## Complex Outcomes of Vegf-C Therapies

Systemic stimulation of fluid drainage and local acceleration of wound healing are two main reasons for the search of the optimal-lymphangiogenic strategies that aim at targeting different compartments of lymphatic circulation, respectively collecting and capillary lymphatics. Despite varying sensitivity and responsiveness of the endothelium in these two compartments of lymphatics,^5^ both strategies take advantage of a single compound, VEGF-C, a factor that has the most restricted pluripotency, particularly the limited potential for collateral activation of blood vessels.^1^

Formation or normalization of collecting vessels increases fluid drainage, which mitigates edema in drained tissue, mechanical tension, and inflammation.^52,87^ This, in turn, can also assist in the resolution of wound healing by withdrawing the tensional stimulus for contractile myofibroblasts.^132^ Increased density of lymphatic capillaries acts locally by accelerating immune cell trafficking to the draining lymph nodes, increasing immune protection of the wound and availability of cytokines and growth factors.^10^ Capillary lymphangiogenesis may be additionally beneficial in the systemic control of blood hypertension^65^ and the reverse cholesterol transport.^26^

Contrary to blood vessels, where drugs are injected directly into the central circulation, lymphatic vessels are targeted indirectly through the release of a compound into the interstitium.^17^ Once in the interstitial space, an active compound accumulates within lymphatic capillaries and stimulates their endothelium. The drug then drains into efferent vessels and becomes condensed, which inevitably affects the endothelium of collecting vessels. Therefore, preserving the treatment specificity for a particular lymphatic compartment is a significant challenge. Indeed, overexpression of VEGF-C in a tumor leads to the hyperplastic changes in the valves of pre-existing collecting vessels, resulting in functional aberrations such as reverse lymph flow.^6^ At least in tumor models, VEGF-C induction of lymphatic abnormalities depends on factors that have not yet been identified.^7^ In a physiological environment, the abnormalities induced by VEGF-C in remote collectors depend on the age of the treated mice. For example, VEGF-C locally expressed in the pulmonary epithelium of newborn mice in a Tet-On–controlled manner induced lymph leakiness, aneurysmal bulges, and abnormal remodeling of valves in the thoracic duct, the central and most remote collecting lymphatic; however, this pathology was not observed when VEGF-C acted on developed lymphatics in 8-month-old mice.^5^ Other reports showed that *in situ* production of VEGF-C specifically activated capillaries but not collecting lymphatics.^2^ Considering this resistance to stimulation and remodeling, it is surprising that VEGF-C promotes regeneration of collectors after the excision of draining lymph nodes in adult mice^133,134^ and pigs.^135^ Some of these discrepancies can be accounted for by poorly defined animal models from which these conclusions are drawn. For example, a recent study questioned the beneficial role of VEGF-C in resolving lymphedema.^136^ In this inflammation-induced lymphedema model, the excision of the axillary lymph node was done under the back skin of the mouse. A leakage from lymphatic collectors, edema, and fibrosis in early and chronic lymphedema correlated with local lymphangiogenesis and VEGF-C levels. However, the specific choice of the readout, that is, the thickness of the back skin is not representative of the pathology of human disease where irreversible subcutaneous fibrosis is the most challenging complication.^59^ Therefore, it is difficult to correlate the reported results with animal models where lymphedema is developed and measured within appendages, for example in a dog leg^97^ or a mouse tail.^98^

A high level of VEGF-C can inflict collateral damage to blood vessels by activating the blood endothelial receptor, VEGFR-2, for which VEGF-C affinity is four times lower than that of the lymphatic VEGFR-3.^137,138^ Indeed, levels of VEGF-C are correlated with blood vessel angiogenesis in breast cancer,^139^ whereas VEGFR-3 is expressed on leaky blood endothelium in the retina of diabetic monkey.^140^ Activation of blood endothelium resulting from the uncontrolled overproduction of adenovirally delivered VEGF-C leads to vessel enlargement and tortuosity^141^ as well as to vascular leakage.^135,142^ Pro-angiogenic properties of VEGF-C were intentionally and successfully used to promote growth of heart collateral vessels in pig myocardial ischemia^3^ and rabbit hindlimb ischemia.^143^ Adenoviral gene delivery is the most effective means of delivering genes *in vivo*, but it comes with a set of limitations, such as immunogenicity and difficulties in optimal dosing of expressed factor.^142^ However, even direct administration of a protein at a low (10-20 ng) dose induced plasma extravasation from blood vessels in normal guinea pig skin that was comparable to the effect of VEGF-A, the most potent inducer of blood vessel permeability.^144^ Similarly, 160 ng of VEGF-C released from a pellet implanted in the cornea stimulated cornea angiogenesis in mice.^4,145^ Even a locally expressed VEGF-C variant with higher VEGFR-3 specificity, VEGF-C156S, was responsible for the acceleration of diabetic wound healing by nonspecific stimulation of blood vessel angiogenesis.^146^ Theoretically, VEGF-C156S, generated by mutagenic modification of VEGF-C, has minimal specificity for VEGFR-2.^147^ However, a recent report revealed that VEGF-C156S is also a weak stimulant of lymphatic vessel growth. The same study also showed that when transiently expressed, the wild-type VEGF-C exerted only a minimal effect on blood vessels.^1^ In support, adenoviral expression of VEGF-C produced no vascular side effects in lymphatic collector excision wounds^1,135^ or after lymph node autologous transplantation.^148^ Finally, implantation of VEGF-C-loaded hydrogel^149^ or single bolus VEGF-C injection,^150^ both at extreme doses of 100 μg, stimulated lymphatic drainage, reduced edema, and normalized tissue architecture without collateral activation of blood vessels.

**Figure 5.**
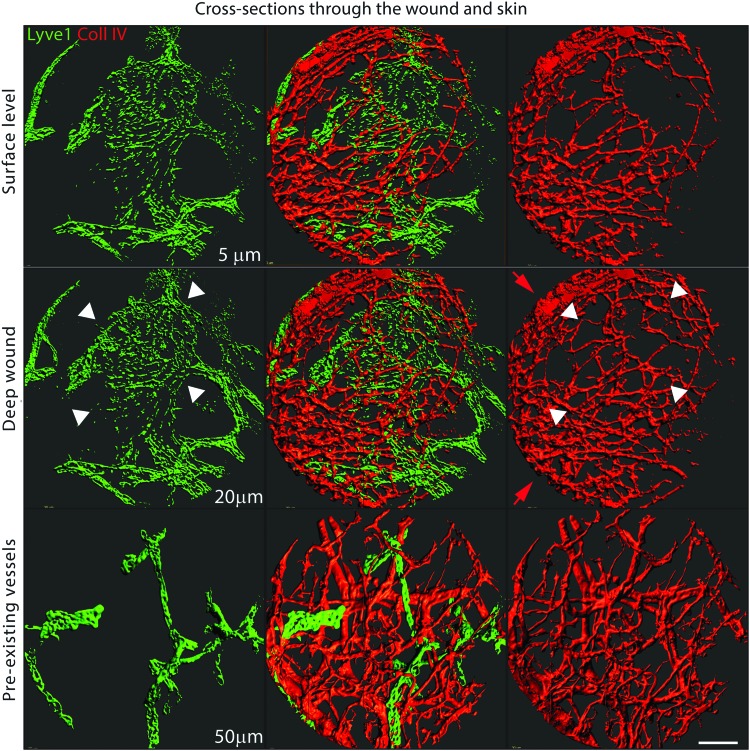
Hyperplasia of capillary lymphatic creates regions with heterogeneous density of blood vessel. A single imaging field of the wound granulation tissue (*top* and *middle rows*) growing from the preexisting vessel (*bottom row*) of the dorsal ear skin. Images show channel-masked maximum intensity projections of optical sections centered at a different depth from the surface of the same wound stained for LYVE-1 (*green*) and collagen IV (*red*) 2 weeks after stimulation with VEGF-C. This staining can distinguish between lymphatics and blood capillaries, as newly formed or VEGF-C-stimulated lymphatics are LYVE1 positive whereas the weak signal from their thin basement membrane is dominated by a signal from the basement membrane of the blood vessel. VEGF-C stimulation resulted in the hyperplastic outgrowth of lymphatic capillaries between the center and the surface of the wound (0–30 μm). The heterogeneity of blood and lymphatic density is highest on the surface of the wound and decreases toward the pre-existing vessels. The *bottom row* shows blood and lymphatic vessels within the pre-existing skin that gave rise to the granulation vessels of the wound. These vessels serve as a reference for the skin vascularity. A large, single-volume lymphatic vessel between the surface (0 μm) and the center of the wound (30 μm) is marked with *arrowheads*. This hyperplastic lymphatic vessel fends off blood vessels from the lymphatic area, concentrating them at the periphery of the hyperplastic vessel (*red arrows*). Bar = 50 μm. To see this illustration in color, the reader is referred to the web version of this article at www.liebertpub.com/wound

**Figure 6.**
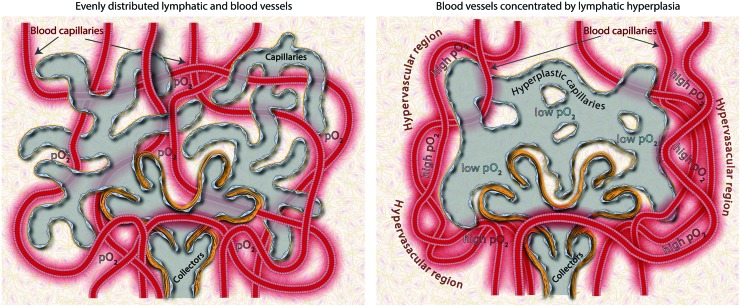
A schematic showing the effect of capillary lymphatic hyperplasia on local density of blood vessels. *Left panel*. Granulation tissue with evenly distributed blood and lymphatic vessels. *Right panel*: Hyperplastic lymphatic capillaries fend off growing blood vessels, creating hypo- and hyper-vascular micro-niches with different access to blood-derived nutrients. pO_2_-normal oxygen pressure. To see this illustration in color, the reader is referred to the web version of this article at www.liebertpub.com/wound

These confounding effects of VEGF-C have been attributed to the high and persistent presence of VEGF-C in the tissue, whereas VEGF-C transiently expressed had only a minimal effect on blood vessels.^2,5^ Even though the temporal and location-dependent control of protein expression *in vivo* reduces the side effects of VEGF-C, reproducible and quantitative delivery of growth factors is possible only with the administration of proteins. In contrast to direct injection of growth factors that generally results in protein clearance within hours,^151^ delayed or controlled release restricts bioavailability of administrated proteins and provides long-lasting local stimulus.^152^ For example, on-demand release of matrix-binding version of blood vascular VEGF-A improved tissue healing, with minimal toxic effect on vascular permeability.^153^ Slow-release systems are also beneficial for VEGF-C therapies. Fibrin-bound VEGF-C released on demand stimulated functional hyperplasia of lymphatic capillaries and had no effect on the number of blood vessels or the morphology of collecting lymphatics (Figs. 5 and 6).^10^ In addition, controlled or delayed release prevents loss of growth factors during the initial post-wounding 3-day lag phase, that is, before the target vessels of granulation tissue develop at the wound periphery.^154,155^ Biodegradable albumin–alginate microparticles release VEGF-C_C152S_, another VEGFR3-specific variant of rat VEGF-C, over the course of weeks. Steep concentration gradients that developed around the particles accelerated rat cardiac lymphangiogenesis and had limited effects on collector remodeling during post-myocardial infarction healing.^156^ Together, the sustained release of growth factors offers an alternative treatment approach that reduces their concentration-dependent pluripotency and, therefore, limits toxicity without altering the effect of the treatment.

Other growth factors, such as VEGF-D^157^ or even typically pro-angiogenic VEGF-A,^158^ are also capable of stimulating lymphangiogenesis. VEGF-A binds VEGFR-2 present on blood and lymphatic vessels and is, therefore, capable of simultaneous stimulation of angio- and lymphangiogenesis. VEGF-D and VEGF-C are bona fide ligands of VEGFR-3, a tyrosine kinase expressed in the adult organism almost exclusively on lymphatic endothelial cells.^159^ VEGF-D is dispensable during development, a property potentially advantageous for its therapeutic applications.^160^ However, in contrast to mouse VEGF-D, which binds only VEGFR-3, human VEGF-D, similar to VEGF-C, binds and activates lymphatic (VEGFR-3) and pan-endothelial (VEGFR2) receptors.^161^ The differences in interspecies receptor specificity of VEGF-D is a significant obstacle, as research on the mouse VEGF-D cannot be translated to humans. Nevertheless, the use of human VEGF-D presents little advantages in therapeutic applications, as it shows even stronger blood vascular effects in pigs and mice as compared to VEGF-C.

## Potential Lymphatic Strategies: Alternative Applications of Lymphatic-Targeted Therapies

### Turning lymphatics into lymphoid-like stroma

**Figure 7.**
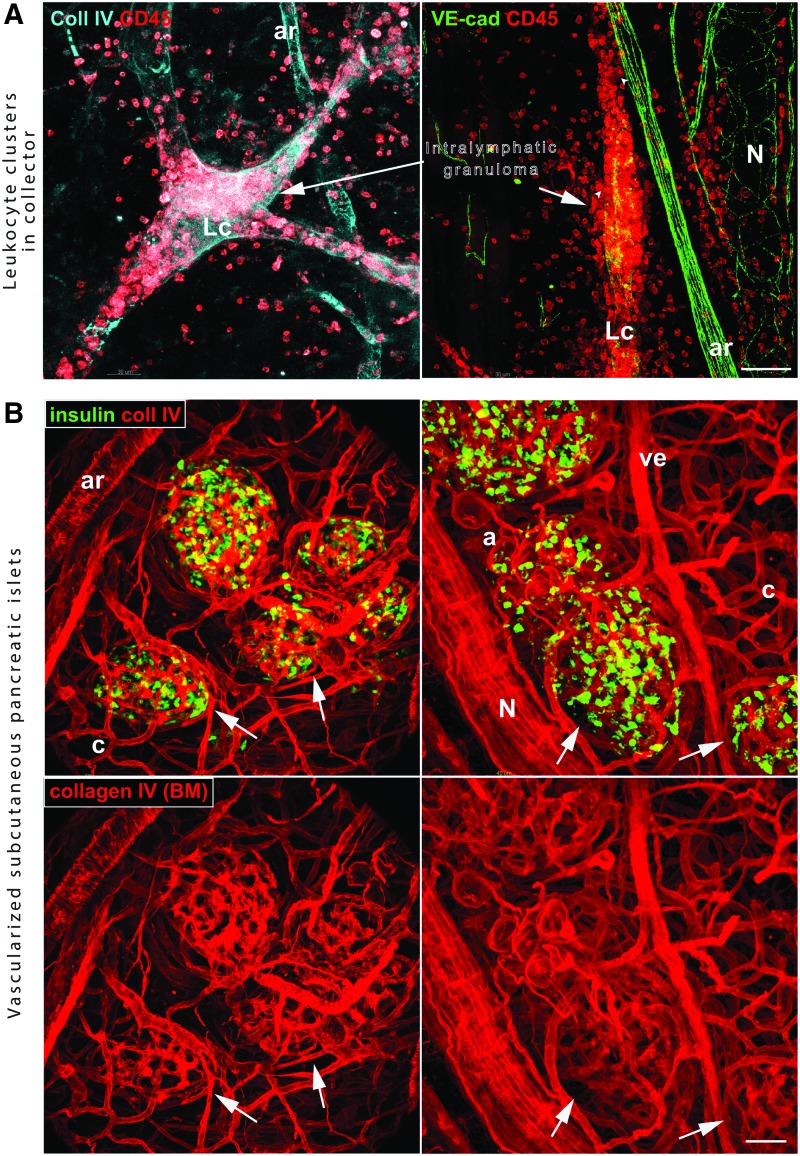
Alternative applications of lymphatic therapies. **(A)** Turning lymphatics into the lymphoid-like stroma. Regenerating lymphatic vessel 8 days after decellularization with photodynamic therapy. *Left* and *right panels* show two examples of lymphatics collectors (Lc) with intralymphatic granuloma composed of lymphocytes and macrophages (*arrows*). **(B)** Hyperplasia of lymphatic capillaries diversifies local density of blood vessels. Hyperplastic lymphatics passively increase blood vessel micro-density that locally enhances vascularization of subcutaneous grafted pancreatic islets. *Left* and *right panels* show two examples of subcutaneously isografted Langerhans islets with beta cells expressing insulin (*green*) 5 weeks after the implantation. Islet vasculature is connected to the host circulation without excessive formation of granulation tissue and fibrotic adipose tissue. Lc = lymphatic collector, *N* = nerve, a = adipocyte cluster, c = blood capillary, ar = artery, ve = vein. Bars = 50 μm. To see this illustration in color, the reader is referred to the web version of this article at www.liebertpub.com/wound

Because of their relative tolerance to morphological and functional abnormalities, lymphatics and capillary vessels, in particular, are well suited for strategies that are aimed at modifying or even changing their function. For example, lymphatic collecting vessels decellularized with anti-lymphatic PDT^44^ become densely populated with lymphocytes and antigen-presenting cells during the regeneration process (Fig. 7A). This ectopic lymphoid-like tissue^162^ formed within otherwise healthy skin has the potential to immunomodulate processes of lymphocyte restimulation.

### Hyperplastic lymphatic capillaries to scavenge growth factors, mitigate fibrosis, and enhance blood vessel density

Hyperplastic lymphatic capillaries induced by local VEGF-C treatment^2,10,117,163^ are tissue-excluding structures not found in healthy tissue. These vessels are formed by non-malignant proliferation of endothelium that sequesters empty volumes of tissue. Hyperplastic capillaries may occupy all available tissue^2^ (Fig. 5), a space significantly larger than could be expected from cells growing in a compact neoplastic manner.^10^

Recently, we showed that these capillaries maintain a functional status, permitting flow, attracting DCs, and cooperating in their ingress into lymphatic circulation.^10^ Most importantly, on-demand released matrix-bound VEGF-C tripled the number of lymphatic endothelial cells, with no effect on the number of blood vessels and only a moderate increase in the granulation tissue thickness. This observation points to some daring conclusions.

First, as long as these lymphatic vessels are functional, they are filled with fluid, which protects the space they enclose from the accumulation of extracellular matrix and, in consequence, excessive fibrosis.^164^ Second, the over-represented lymphatic endothelial cells can shield blood vessels from superfluous stimulation and limit their remodeling by scavenging their common growth factors, such as VEGF-A. Practically, a physiological buffering of the pro-angiogenic environment with peritumoral hyperplastic lymphatics could normalize and reprogram dysfunctional tumor blood vessels.^165^ Lastly, with a minimal change in the volume of granulation tissue and the same number of blood vessels as in a non-treated wound, the exclusion of large portions of extracellular space by hyperplastic lymphatics should effectively diversify the micro-density of blood vessels, creating hypo- and hypervascular zones (Figs. 5 and 6). Therefore, a local increase in tissue vascularity could be achieved without potent angiogenic stimuli and with minimal formation of excessive granulation tissue and subsequent fibrosis. In consequence, a hypervascular micro-niche can more efficiently vascularize oxygen-hungry endocrine grafts (Fig. 7B), where poor vascularity of a subcutaneous fibrotic transplant and its consequent inadequate oxygenation is the primary obstacle for subcutaneous grafting of small tissues such as pancreatic islets.^166^ The influence of hyperplastic lymphatic on tissue state may extend beyond the healing or inflammatory phase as in contrast to blood vessels that are normalized and pruned after inflammatory stimuli subsided, hyperplasia of lymphatics is persistent.^2,117^

## Summary

Contrary to expectations, the current research shows that organizational simplicity and capillary tolerance for functional flows, features inherent to the lymphatic system, do not necessarily translate into straightforward treatment applications. Lack of growth factors that are unique to specific lymphatic compartments increases the risk of not only lymphatic abnormalities but also off-target activation of the blood vasculature. The desired effect on lymphatic function might be more readily achieved with control-released VEGF-C delivery methods. Finally, lymphatics might be targeted to exploit their functional redundancy and nature of volume-sequestering structures and to reshape their biology to gain new functions such as enhanced antigen presentation or increased vascular density.

Take-Home Messages

• The relevance of new lymphatic functions has not been verified in known human pathologies, and the complete etiology of secondary lymphedema is unknown.
• Capillary and collecting lymphatics serve different functions, with no known diseases ascribed to capillary abnormalities.
• Physiological functionality is a unique feature of lymphatic hyperplasia, a persistent outgrowth of lymphatic capillaries.
• The outcome of VEGF-C treatment on lymphatics must be separated from its effect on blood vessels.
• Lymphatic specificity of VEGF-C can be achieved with the delayed or controlled release of VEGF-C.

## About the Author

**Witold W. Kilarski, PhD,** is a Research Assistant Professor at the University of Chicago, currently working on the mechanism of lymphatic filaria infection and application of anticoagulant properties of lymphatic vessels. He graduated from the Cracow Jagiellonian University and continued his education at the Uppsala University where his Ph.D. work culminated in the discovery of a non-developmental mechanism of blood vessel growth. During his first postdoc at the University of Bordeaux, he was engaged in a transcriptomic and proteomic analysis of diseased blood vasculature. As a research fellow at École Polytechnique Fédérale de Lausanne in Switzerland, he worked on physiological aspects of lymphatic vessels and developed an intravital imaging assay to study blood vessel and lymphatic function in small tissue transplants. His research interests focus on manipulation of the blood and lymphatic vessel microenvironment for optimal vascularization and immunoprotection of small endocrine tissue grafts.

## Abbreviations and Acronyms

BM: basement membrane
DC: dendritic cell
CNS: central nervous system
HDL: high-density lipoproteins
HSPG: heparan-sulfate proteoglycan
PDT: photodynamic therapy
VEGF: vascular endothelial growth factor
VEGFR: vascular endothelial growth factor receptor
